# Analysis of spatial and temporal characteristics of carbon emission efficiency of pig farming and the influencing factors in China

**DOI:** 10.3389/fpubh.2023.1073902

**Published:** 2023-01-26

**Authors:** Hongpeng Guo, Shi Li, Chulin Pan, Shuang Xu, Qingyong Lei

**Affiliations:** College of Biological and Agricultural Engineering, Jilin University, Changchun, China

**Keywords:** carbon emission efficiency, SBM model, distribution characteristics of carbon emission efficiency, Tobit model, factors affecting carbon emission efficiency

## Abstract

Pig farming has been a crucial contribution to China's food security although intestinal fermentation and its excrement during pig breeding are major sources of greenhouse gas emissions. In this paper, we measured the carbon emission efficiency of pig farming in 30 provinces (autonomous regions and municipalities) from 2010 to 2020 by using the non-expected output Slack-Based Measure (SBM) model and analyzed the spatial characteristics of the carbon emission efficiency of pig farming in China. We also examined and analyzed the factors influencing the carbon emission efficiency of pig farming by using the limited dependent variable model (Tobit). The results show that: the carbon emission efficiency of pig farming in China shows an M-shaped upward trend over time by comparing the carbon emission efficiency longitudinally during the study period and the carbon emission efficiency of pig farming shows a decreasing trend in the east, central and west regions of China by comparing the carbon emission efficiency of different regions horizontally. It's also shown that regions with low- and extremely-low-efficiency transfer from the east to the central and west regions and the central and regions with high-efficiency transfer to the east. The regression analysis of the factors influencing the carbon emission efficiency of pig breeding shows that the comparative advantage of the pig industry and transportation accessibility is positively correlated with the carbon emission efficiency of pig breeding, whereas the proportion of food resources and market scale is negatively correlated with the carbon emission efficiency of pig breeding. At the same time, the production layout index has no significant influence on the carbon emission efficiency of pig breeding. The research results provide a theoretical basis for regional differentiation of carbon emission management from pig farming, optimizing the layout of the pig industry and reducing environmental pollution.

## 1. Introduction

Carbon emissions from livestock and poultry farming have been one of the most significant sources of greenhouse gas emissions in China (1). Statistics from the Food and Agriculture Organization of the United Nations (FAO) show that pigs, cattle, sheep, and poultry account for 18% of overall greenhouse gas emissions. China is not only a major pig-producing country but also a major pork-consuming country. In China, both pig rearing and pork consumption account for half of the world's total. The pig farming industry pollutes water, air, and soil, making the rural ecological environment more fragile and limiting the sustainable development of the industry (2). The carbon emission from pig farming has become the second largest type of carbon emission from livestock farming in China, second only to that from cattle farming. The carbon emission from pig farming has become one of the major difficulties in the management of agricultural surface pollution in China (3). It is very important to get higher profits with lesser carbon emissions and carbon emission efficiency has been used to measure this index. By referring to other scholars' definitions of carbon emission efficiency (4–6), this paper defines the carbon emission efficiency of pig breeding as low carbon emission to obtain maximum benefits in the process of pig breeding with certain input factors.

Livestock production accounts for a significant share of the global greenhouse gas (GHG) balance, including carbon dioxide (CO_2_), methane (CH_4_), ammonia (NH_3_), and nitrous oxide (N_2_O) released into the atmosphere (7). There are many calculation and assessment methods to study carbon emission efficiencies, such as the stochastic frontier model, regression model, data envelopment analysis (DEA) method, and unexpected output (Slack-Based Measure) model (8–11). Most studies have adopted the unexpected output (Slack-Based Measure) model to calculate carbon emission efficiency. For example, Zhao et al. (12) used the Slack-Based Measure (SBM) model to measure the agricultural eco-efficiency of 31 provinces in China during 2010–2019 and analyzed the spatial-temporal differences (12). Wang and Du (13) also used the Slack-Based Measure (SBM) model to measure the carbon emission efficiency and environmental efficiency of 14 cities in Hunan Province in China from 2010 to 2016 and analyzed the spatial differences.

Domestic and foreign scholars have carried out a large number of studies on the influencing factors of carbon emissions from pig farming. Due to the differences in the farming environment, mode of farming as well as fecal waste treatment methods at home and abroad, the influencing factors of carbon emissions from pig farming also vary. Some developed countries mainly adopt intelligent management systems, automatic phased feeding systems, pig farm environmental monitoring systems, and other modern techniques to carry out real-time feeding and testing of pig breeding, reducing carbon emissions through the improvement of technical means (14). Domestic scholars mainly study the influencing factors of carbon emissions from animal husbandry, including economic development, breeding scale, industrial structure, agricultural technical conditions, education level, scales of the agricultural labor force, urbanization level, etc., (15–19).

With the current national emphasis on environmental pollution control, most scholars have measured and evaluated the carbon emission efficiency of agriculture and animal husbandry in the context of environmental pollution (20–22). However, scholars have not studied the carbon emission efficiency of a single industry, and the relationship between the change in pig industry layout and the carbon emission efficiency of pig farming and the influencing factors are still unknown. Based on the above studies, this paper uses the non-expected output Slack-Based Measure (SBM)model to measure the carbon emission efficiency of pig breeding in 30 provinces (autonomous regions and municipalities) in China and combines the Tobit model to test and analyze its influencing factors. It provides the government with theoretical references and policy guidance on the layout of the pig industry and how to improve the carbon emission efficiency of pig farming to reduce pollution.

## 2. Materials and methodology

### 2.1. Spatial and temporal characteristics of carbon emission efficiency of pig farming

#### 2.1.1. Data sources

The data required for this paper were obtained from the 2010–2020 China Statistical Yearbook^1^ and the China Rural Statistical Yearbook^2^, as well as provincial and municipal statistical yearbooks and statistical bulletins on national economic and social development. Due to the unavailability of data, Hong Kong, Macau, Tibet and Taiwan have not been included in the study.

#### 2.1.2. Indicator construction

Through reviewing a large amount of literature, this study selected pig farming capital, and labor (23) as input indicators, pig production value (1), and carbon emission (24) as desired output indicators and non-desired output indicators, respectively (Table 1).

**Table 1:** Carbon emission efficiency measurement index of pig farming.

**Category**	**Indicators**	**Metrics**
Input elements	Capital Investment Labor input	Pig farming cost Number of employees in the pig industry
Output elements	Expected output Non-desired outputs	Pig production value Carbon emissions from pig farming

#### 2.1.3. Costs of pig farming

The total costs of pig farming in each province and city were obtained by calculating the product of the cost per pig farmed and the annual pig slaughter volume in each province and city by reviewing the literature and referring to the China Rural Statistical Yearbook^3^ and the statistical yearbooks of each province and city.

#### 2.1.4. Number of employees in the pig industry

Due to the lack of accurate data on the number of employees in the pig industry, by reviewing the literature, we refer to the methods and ideas of Zhang et al. (23) to calculate the amount of investment in fixed assets and the number of employees in the livestock industry. The formula for calculating the number of employees in the pig industry is shown below.


(1) $$\begin{array}{l} PE_{k}^{t}=XE_{k}^{t}\frac{POV_{k}^{t}}{XOV_{k}^{t}} \end{array}$$

In the formula: *PE* is the number of employees in the pig industry, *XE* is the number of employees in the livestock industry, *POV* is the output value of pigs, *XOV* is the output value of livestock, k is the region, and t is the period.

#### 2.1.5. Carbon emissions from pig farming

After reviewing the literature, it is found that the carbon emissions of pig farming mainly come from methane (CH_4_) produced by the intestinal fermentation process of pigs, CH_4_ (1 t CH_4_ = 6.82 carbon), and nitrous oxide (N_2_O, 1 t = N_2_O = 81.27 t carbon) from manure emissions. Referring to the IPCC Greenhouse Gas Emissions Inventory Guidelines 2019, carbon emissions are calculated as follows:


(2) $$\begin{array}{l} C=\overset{1}{\underset{i=1}{\sum }}C_{k}^{t}=\overset{1}{\underset{i=1}{\sum }}\left[6.82\times {\text{λ}}_{k}^{t}\times \left(\mu_{i}+\nu_{i}\right)+81.27\times {\text{λ}}_{k}^{t}\times \omega_{i}\right] \end{array}$$

Where: *C* is the total carbon emission, $C_{k}^{t}$ is the carbon emission from pig farming in year t in region *k*, ${\text{λ}}_{k}^{t}$ is the average feeding capacity of pigs in year t in region *k* [see Equation (4) for the calculation of the average feeding capacity], *μ*_*i*_, *v*_*i*_, *ω*_*i*_ are the CH_4_ and N_2_O emission coefficients of pigs, and by referring to the IPCC Guidelines for Greenhouse Gas Emission Inventories 2019, we know that the emission coefficient of CH_4_ from enteric fermentation of pigs (*μ*_*i*_) is 1, the emission coefficient of CH_4_ from manure emissions from CH_4_ (*v*_*i*_) is 3.5 and N_2_ O (*ω*_*i*_) is 0.53, respectively.


(3) $$\left\{ \begin{array}{l} {\text{λ}}_{k}^{t}=\phi_{k}^{t}\times \frac{\varphi_{k}^{t}}{365}\;\gamma \geq 1\\ \;{\text{λ}}_{k}^{t}=\frac{\tau_{k}^{t}+\sigma_{k}^{t}}{2}\;\gamma <1 \end{array}\right.$$

Where: λ is the average stocking, Φ is the average annual growth cycle, ϕ is the annual slaughter, *γ* is the slaughter rate (where the slaughter rate of pigs and poultry is ≥ 1, and the average annual growth cycle is 200 d and 55 d, respectively), *τ* is the stocking at the end of the previous year, *σ* is the stocking at the end of the current year, *k* is the region, and *t* is the period.

#### 2.1.6. Non-desired output SBM model

Modern production methods have increased labor productivity, which not only facilitates the increasing trade and economic activities between countries, but it also improves people's living standards with the privilege of having abundant and cheap industrial products. At the same time, industrial production inevitably produces large amounts of pollutants such as wastewater, waste gas, and solid waste, which are commonly referred to as undesired outputs.

Furthermore, it leads to a series of problems such as haze and global warming. Therefore, green production methods which reduce waste have become a crucial goal in every production area. If non-desired outputs are being considered, we do not want to produce more industrial waste, no matter what the input is. Therefore, the most efficient production method in today's society is the green production method, producing more desired outputs with fewer inputs as well as fewer undesired outputs. Tone proposed the undesired output Slack-Based Measure (SBM) model in 2003. The model is based on the Slack-Based Measure (SBM) model proposed by Tone in 2001 (23).

Suppose there are *n* decision units, each of which contains three elements: three vectors of inputs *X*, desired outputs *Y*^*g*^ and non-desired outputs (production emissions such as wastewater, CO_2_, soot, etc.) *Y*^*b*^, which can be expressed as


(4) $$\begin{array}{l} \begin{array}{c} X=\left[x_{1},\ldots ,x_{n}\right]\in R^{m\times n}\\ Y^{g}=\left[y_{1}^{g},\ldots ,y_{n}^{g}\right]\in R^{s_{1}\times n}\\ Y^{b}=\left[y_{1}^{b},\ldots ,y_{n}^{b}\right]\in R^{s_{2}\times n} \end{array} \end{array}$$

Where: *X, Y*^*g*^, *Y*^*b*^ > 0, *R* is the set of real vectors, *m, s*_1_, *s*_2_ are the number of factors of input, desired output, and non-desired output, respectively. The SBM model for non-desired outputs can be expressed as


(5) $$\left\{ \begin{array}{l} \;\rho^{*}=\frac{1-\frac{1}{m}\sum_{j}^{m}\frac{S_{i}^{-}}{x_{i_{0}}}}{1+\frac{1}{s_{1}+s_{2}}\left(\sum_{r=1}^{s_{1}}\frac{S_{r}^{g}}{y_{r}^{g_{0}}}+\sum_{r=1}^{s_{2}}\frac{S_{r}^{b}}{y_{r}^{b_{0}}}\right)}\\ s.t.\;x_{0}=X\text{λ}+s^{-},y_{0}^{g}=Y^{g}\text{λ}-s^{g},y_{0}^{b}=Y^{g}\text{λ}+s^{b}\; \end{array}\right.$$

In this model ρ^*^ is the carbon emission efficiency value, and *s*^−^, *s*^*g*^ and *s*^*b*^ are the slack amounts of input, desired output, and non-desired output, respectively. When ρ^*^ = 1, the decision unit is valid, i. e., there is a Pareto optimum; when ρ^*^ > 0~ < 1, it is in an invalid state, and the efficiency can be improved by optimizing the input and output. Efficiency is specifically divided into four levels: very low efficiency (0 < ρ^*^ ≤ 0.3), low efficiency (0.3 < ρ^*^ ≤ 0.6), medium efficiency (0.6 < ρ^*^ ≤ 0.9), and high efficiency (ρ^*^ > 0.9) (25).

#### 2.1.7. Carbon emission efficiency values

In this paper, Equations (1)–(5) and Matlab software are used to measure the carbon emission efficiency values of 30 provinces (autonomous regions and municipalities) in China from 2010 to 2020, and the results are shown in Table 2.

**Table 2:** Carbon emission efficiency values of pig farming in 30 provinces, cities, and autonomous regions nationwide, 2010–2020.

**Region**	**2010**	**2011**	**2012**	**2013**	**2014**	**2015**	**2016**	**2017**	**2018**	**2019**	**2020**
Beijing	0.862	0.847	0.905	0.938	0.882	0.776	0.438	0.794	0.953	0.114	1.000
Tianjin	0.413	1.000	1.000	1.000	1.000	1.000	1.000	1.000	1.000	1.000	1.000
Hebei	0.397	0.447	0.408	0.468	0.437	0.470	0.296	0.298	0.465	0.309	0.467
Shanxi	0.414	0.370	0.307	0.304	0.296	0.323	0.240	0.296	0.291	0.214	0.379
Inner Mongolia	0.398	0.408	0.377	0.439	0.447	0.485	0.279	0.416	0.399	0.417	0.483
Liaoning	0.472	0.701	0.536	0.617	0.534	0.544	0.288	0.315	0.318	0.183	0.129
Jilin	1.000	0.715	0.718	0.650	0.716	0.727	1.000	0.445	0.554	0.274	0.414
Heilongjiang	1.000	1.000	1.000	1.000	1.000	1.000	1.000	1.000	0.893	1.000	1.000
Shanghai	1.000	1.000	1.000	1.000	1.000	1.000	1.000	1.000	1.000	0.508	1.000
Jiangsu	0.424	0.539	0.537	0.592	0.531	0.545	0.347	0.716	0.844	0.449	0.608
Zhejiang	0.418	0.698	0.635	0.693	0.690	0.839	0.828	1.000	1.000	0.585	0.833
Anhui	0.462	0.458	0.453	0.439	0.429	0.471	0.493	1.000	0.706	0.314	0.747
Fujian	0.522	0.652	0.594	0.633	0.623	0.687	0.476	0.703	0.903	0.516	1.000
Jiangxi	0.346	0.408	0.380	0.415	0.422	0.467	0.196	0.192	0.207	0.218	0.264
Shandong	0.556	0.635	0.582	0.604	0.579	0.657	0.532	0.615	0.677	0.446	0.382
Henan	0.322	0.427	0.411	0.422	0.421	0.448	0.238	0.280	0.479	0.236	0.318
Hubei	1.000	0.553	0.472	0.535	0.563	0.596	0.551	0.577	0.783	0.491	0.679
Hunan	0.315	0.385	0.338	0.370	0.335	0.383	0.246	0.283	0.376	0.360	0.591
Guangdong	0.340	0.410	0.410	0.443	0.431	0.449	0.215	0.418	0.467	0.329	0.373
Guangxi	0.309	0.317	0.348	0.330	0.308	0.343	0.213	0.436	0.360	0.259	0.189
Hainan	0.492	0.736	0.680	0.673	0.630	0.788	0.766	0.998	0.984	1.000	0.780
Chongqing	0.188	0.199	0.237	0.271	0.239	0.238	0.166	0.178	0.206	0.214	0.412
Sichuan	0.332	0.357	0.333	0.370	0.353	0.460	0.200	0.227	0.266	0.324	0.432
Guizhou	0.184	0.189	0.191	0.220	0.254	0.377	0.550	1.000	1.000	0.506	0.386
Yunnan	0.241	0.266	0.303	0.369	0.322	0.352	0.417	0.430	0.504	0.257	0.370
Shaanxi	1.000	0.615	0.602	0.639	0.584	0.649	1.000	0.819	0.787	0.457	0.570
Gansu	0.288	0.261	0.241	0.266	0.273	0.306	0.229	0.226	0.282	0.197	0.198
Qinghai	0.231	0.285	0.261	0.303	0.294	0.256	0.204	0.344	0.441	0.250	0.209
Ningxia	0.283	0.293	0.328	0.342	0.325	0.335	0.377	0.327	0.414	0.241	0.328
Xinjiang	0.515	0.578	0.346	0.493	0.647	0.806	0.590	0.478	0.750	0.524	0.847

### 2.2. Analysis of factors affecting carbon emission efficiency of pig farming

#### 2.2.1. Data sources

The data required for this paper are obtained from the carbon emission efficiency values calculated in the previous sections, those from the China Statistical Yearbook and the China Rural Statistical Yearbook for 2010–2020, as well as from provincial and municipal statistical yearbooks and statistical bulletins on national economic and social development. Due to the lack of availability of data, Hong Kong, Macau, Tibet and Taiwan have not been included in the study.

#### 2.2.2. Introduction to the Tobit model

The national carbon emission efficiency values from pig farming obtained from the efficiency evaluation are all > 0, which are truncated data. The Tobit model is a model in which the dependent variable is continuous but subject to some restrictions on its value. Therefore, it's also known as a restricted dependent variable model. The Tobit model focuses on the analysis of how continuous variables change under a certain choice of behaviors. The general form of the model is shown below:


(6) $$\begin{array}{c} y_{i}=\beta^{T}X_{i}+\varepsilon_{i},i=1,2,\ldots ,n,\varepsilon_{i}\sim N\left(0,\sigma^{2}\right)\\ \;\{\begin{array}{cc} y^{*}\leq 0, & y_{i}=0\\ y^{*}\geq 0, & y_{i}=y^{*} \end{array} \end{array}$$

In Equation (6), *y*_*i*_ is the explained variable, *X*_*i*_ is the explanatory variable, *β*^*T*^ is the parameter vector, and ε_*i*_ denotes the random error term of the model equation that follows a normal distribution. Tobit model is an intercept regression model, where the explanatory variable *X*_*i*_ takes actual observations and the explained variable *y*_*i*_ takes values in a restricted manner: when y^*^ ≥ 0, *y*_*i*_ takes actual observations, and when y^*^ ≤ 0, *y*_*i*_ takes values of 0.

#### 2.2.3. Selection of indicators

For pig farming, the change of various influencing factors triggers the change of production layout through the change of the number of pigs in each region, which will lead to the change in the carbon emission efficiency of pig breeding in each region. Microscopically, the change in pig farming layout in China is formed by the change in the scale of the feeding of many pig farmers. At the same time, the change in pig farming layout will also lead to the industrial shift of pig farming and thus the change in carbon emission efficiency in each region. Therefore, any change of factors leading to the change of pig farmers' feeding scale will become a factor affecting the change of pig farming layout in China. The formation of the regional layout of pigs is the product of natural, economic, and social interaction at a certain stage of development. According to the theory of agricultural regional factor formation developed based on location theory, there are many factors affecting the carbon emission efficiency of pig farming. This study selects the carbon emission efficiency of pig farming as the dependent variable and, by referring to the research results of other scholars, we select the proportion of grain resources (26), production layout index (27), market size (26), transportation accessibility (28) and comparative advantage of pig industry (2) as independent variables. Hence we construct the model as below:


(7) $$\begin{array}{c} PE_{k}^{t}=\alpha_{k}^{t}+\beta_{1}LS_{k}^{t}+\beta_{2}PLI_{k}^{t}+\beta_{3}SG_{k}^{t}+\beta_{4}BS_{k}^{t}+\beta_{5}JT_{k}^{t}+\varepsilon_{k}^{t} \end{array}$$

Where: *PE* is the carbon emission efficiency generated by pig farming, *LS* is the share of food resources, *PLI* is the production layout index, *SG* is the market size, *BS* is thcomparativedvantage, *JT* is the transportation accessibility, *β*_1_ ~ *β*_5_ are the estimation coefficients, ε is the disturbance term, *k* is the region, and *t* is the period.

#### 2.2.4. Interpretation of indicators

##### 2.2.4.1. Share of food resources

Grain, as the main type of feed for pigs, has an important impact on pig farming. The amount of grain production directly determines whether farmers have enough grain to convert into feed grain for pig farming in addition to their subsistence needs. Hence, it has an impact on the behavioral choice of whether to engage in pig farming. Generally speaking, farmers who have resources of grain to convert into feed grain will continue or expand pig farming scale, whereas farmers who do not have grain to convert into feed grain will face two choices of either withdrawing from production or purchasing feed grain. The latter option translates to increased breeding costs (29). Therefore, the abundance or scarcity of grain resources, especially corn and soybean resources, directly affects the pig farming scale of farmers.

##### 2.2.4.2. Production layout index

The production layout index can better reflect the regional distribution and scale of pig farming, and the regional distribution and scale of pig farming affect the change of carbon emission efficiency in each region. In this paper, the production layout index is measured by the proportion of pig farming in each region to the national pig farming in that specific year (27).

##### 2.2.4.3. Market size

Supply and demand theory suggests that an increase in demand leads to an increase in supply, which in turn makes the equilibrium quantity rise and demand is the most direct factor affecting the change in supply. The market size has an important impact on the distribution of live pig farming (27). The larger the market size is, the more people will consume pigs and the more pigs are being kept in the nearby area. The increase in the number of pigs kept will also have an impact on the carbon emission efficiency of the area.

##### 2.2.4.4. Comparative advantage of the pig industry

If pig farming in each region has obvious advantages over agriculture, it will push more farmers and enterprises to enter the pig-producing industry. that the reality is that major agricultural and animal husbandry enterprises are entering the pig industry and expanding their production capacity drastically. The more obvious the comparative advantage of pig breeding, the greater the potential and space for pig farming (30). The greater the pig farming potential and space, the more likely other regions will have a large pig farming potential and space for transfer, and the carbon emission efficiency will change accordingly.

##### 2.2.4.5. Transportation accessibility

The distance between the place of consumption and the origin of pigs determines the source of pork in that individual market. Without considering the differences in pork quality, transportation costs become the most substantial cost factor when the differences in pig farming costs between regions are small. In addition to the distance from the market, the transportation cost is largely related to the transportation systems and conditions of the region. The more developed the transportation systems and conditions are, the easier it is for pig farming to form a scale, which generates more carbon emissions, and, as a consequence, the carbon emission efficiency will change (26).

#### 2.2.5. Variable description

To interpret the model variable data more effectively, this study provides descriptive statistics on the variables and explains the measures of the independent variables in Table 3.

**Table 3:** Descriptive statistics of variables.

**Variables**	**Observed values**	**Average value**	**Standard deviation**	**Minimum value**	**Maximum value**	**Metrics**
PE	330	0.5200	0.25600	0.11400	1.00000	Carbon efficiency of pig farming
LS	330	0.33200	0.02800	0.00434	0.11410	Grain production by region/total national grain for the year
PLI	330	2.05100	3.22800	0.00033	18.6868	Pig slaughter volume by region/national pig slaughter volume of the year
SG	330	0.33200	0.02000	0.00420	0.08940	Total population by region/year-end national population of the same period
BS	330	0.38300	0.16100	0.02850	0.75670	Output value of pig industry by region/total output value of livestock industry
JT	330	0.96200	0.51500	0.08900	2.23400	Total road and rail mileage by region/land area

## 3. Results

### 3.1. Spatial and temporal characteristics of carbon emission efficiency of pig farming

#### 3.1.1. Analysis of time-series characteristics of carbon emission efficiency of pig farming in China

**Figure 1:**
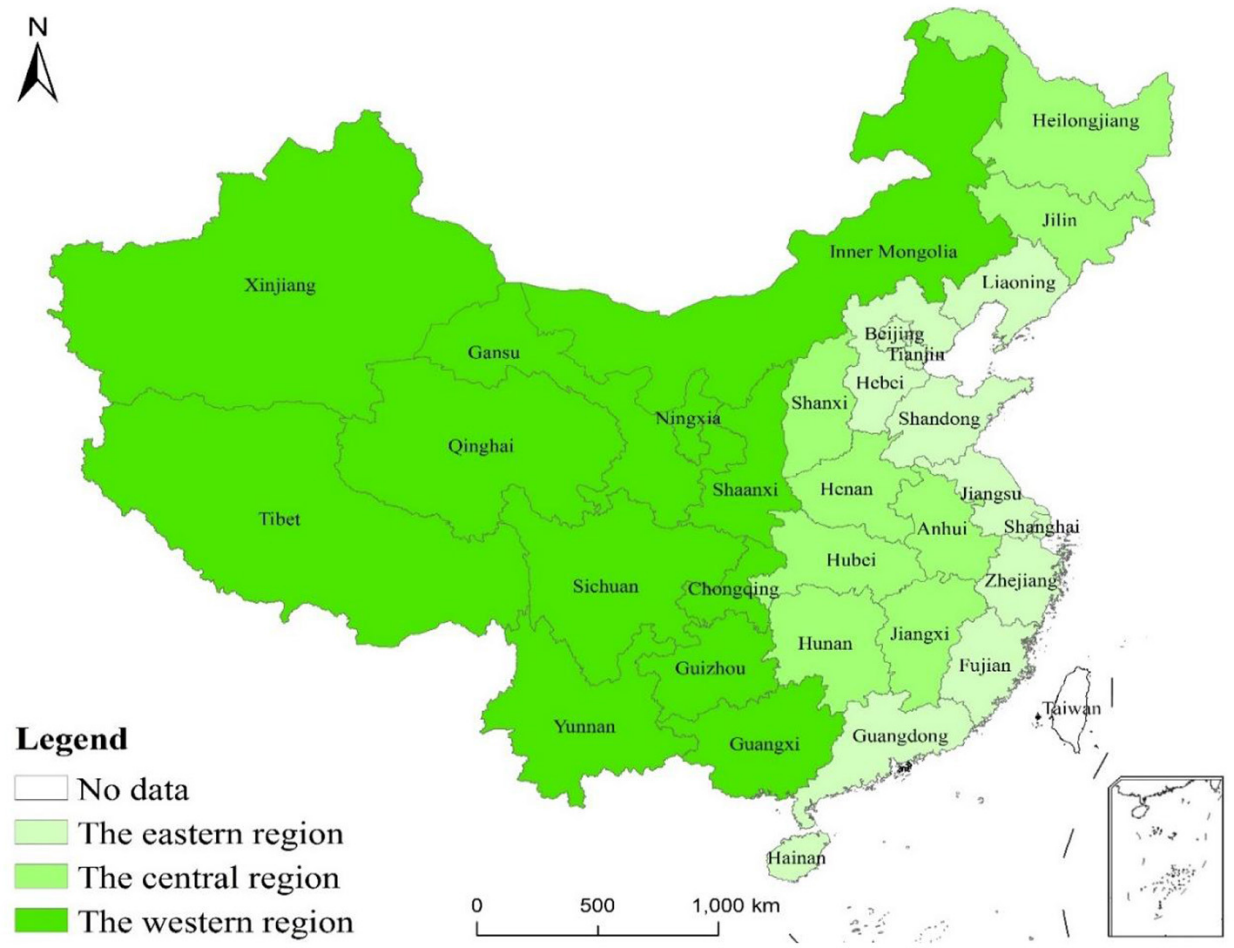
Spatial characteristics of the three major regions in China.

Due to the vast geographic landscape of China, there are obvious differences in resource endowment and support policies in pig farming across the country. To analyze the spatial and temporal differences in the carbon emission efficiency of pig farming in different regions, the 30 provinces (autonomous regions and municipalities) in China were divided into three major regions, eastern, central, and western regions for analysis, as shown in Figure 1.

**Figure 2:**
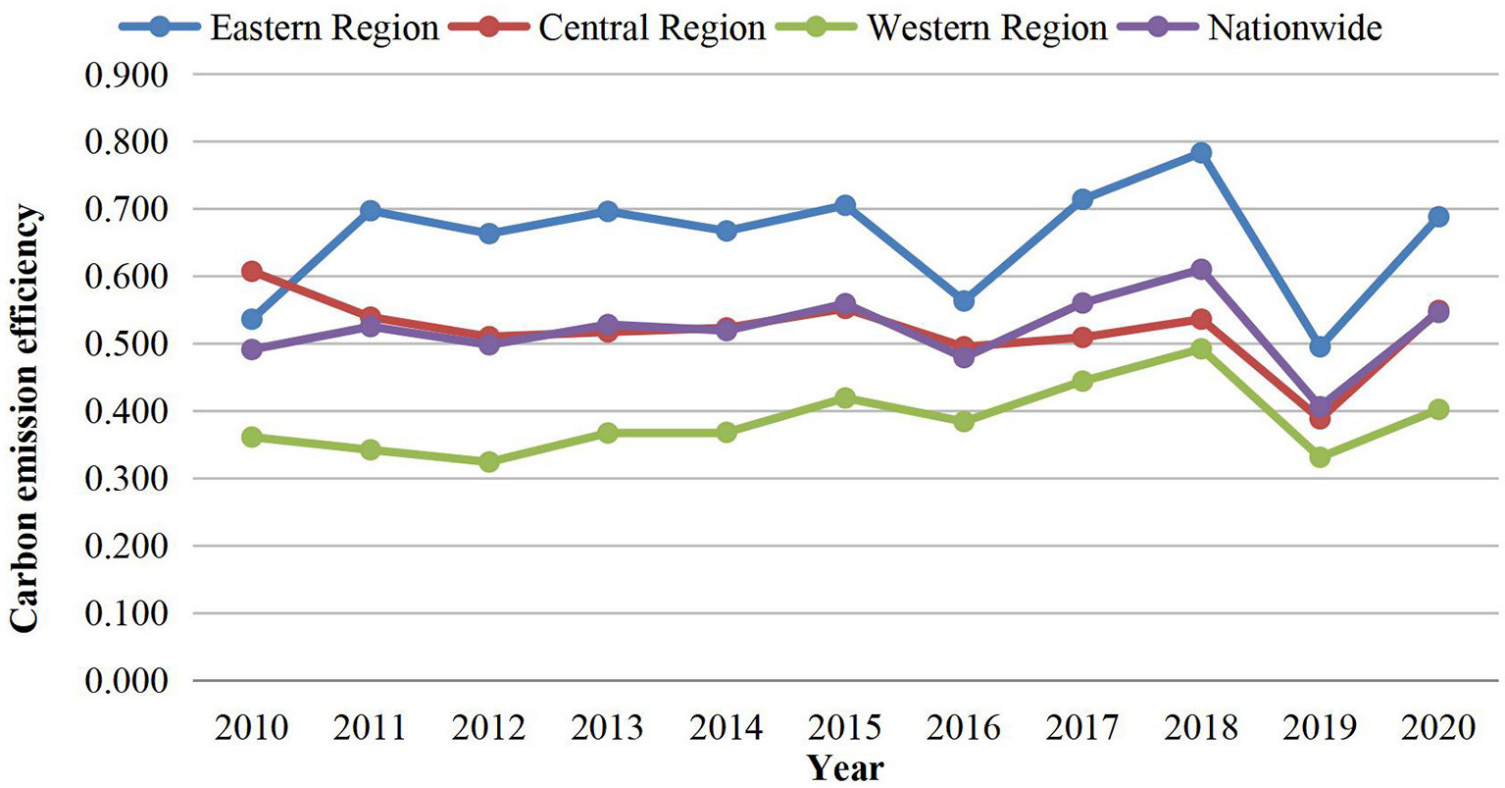
The trend of carbon emission efficiency of pig farming in China, 2010–2020.

To better analyze the differences and trends of carbon emission efficiency of pig farming in different regions from 2010 to 2020, the average values of carbon emission efficiency in the eastern region, central region, western region, and the whole country were calculated. The results are shown in Table 4. A line graph was also drawn, which is shown in Figure 2.

**Table 4:** The average value of carbon emission efficiency of pig farming in China, 2010–2020.

**Region**	**2010**	**2011**	**2012**	**2013**	**2014**	**2015**	**2016**	**2017**	**2018**	**2019**	**2020**
Eastern region	0.536	0.697	0.663	0.696	0.667	0.705	0.563	0.714	0.783	0.495	0.688
Central region	0.607	0.539	0.510	0.517	0.523	0.552	0.495	0.509	0.536	0.388	0.549
Western region	0.361	0.342	0.324	0.367	0.368	0.419	0.384	0.444	0.492	0.331	0.402
Nationwide	0.491	0.525	0.498	0.528	0.519	0.559	0.479	0.560	0.610	0.406	0.546

Looking into three different regions, the carbon emission efficiency value of pig farming shows a trend of “East > Central > West.” The efficiency value of the eastern region increased from 0.536 in 2010 to 0.688 in 2020, with an increase of 28.4% realizing the transformation from a low to medium efficiency zone. The carbon emission efficiency value of the central region decreased from 0.607 in 2010 to 0.549 in 2020, with a decline of 10% changing from a medium to a low-efficiency zone. Carbon emission efficiency in the western region, on the other hand, increased from 0.361 in 2010 to 0.402 in 2020, with an increase of 11.4%. It remains in the low-efficiency zone.

From 2010 to 2020, the carbon emission efficiency of pig farming in China generally showed an M-shaped upward fluctuating trend. Although the nationwide carbon emission efficiency was still at a low-efficiency level in 2020, the carbon emission efficiency value has increased from 0.491 to 0.549. The value was close to the medium efficiency level, indicating that the ratio of input to output is more harmonious, which improves the carbon emission efficiency. From 2010 to 2014, the carbon emission efficiency of pig farming in China showed a relatively steady change and fluctuated up and down around 0.5. The first high and first low-value points of “M” appeared in 2015 and 2016 respectively but the difference between the high and low-value points of efficiency was not significant. From 2016 to 2018, the carbon emission efficiency of Chinese pig farming showed a significant upward trend and reached a medium efficiency level in 2018. The efficiency value dropped deeply in 2019 followed by a rebound in 2020, probably due to the downward adjustment of inputs and outputs caused by the epidemic.

The carbon emission efficiency values of the three regions from 2010 to 2020 were generally consistent with the national trend. The carbon emission efficiency values of the eastern region were at the medium efficiency level in all years except for 2010 and 2019 when they were at a low-efficiency level. The central region only reached a medium efficiency level in 2010 with all the other years at a low-efficiency level. The carbon emission efficiency of the western region was at a low-efficiency level during the period of study.

#### 3.1.2. Spatial characteristics analysis of carbon emission efficiency of pig farming in China

**Figure 3:**
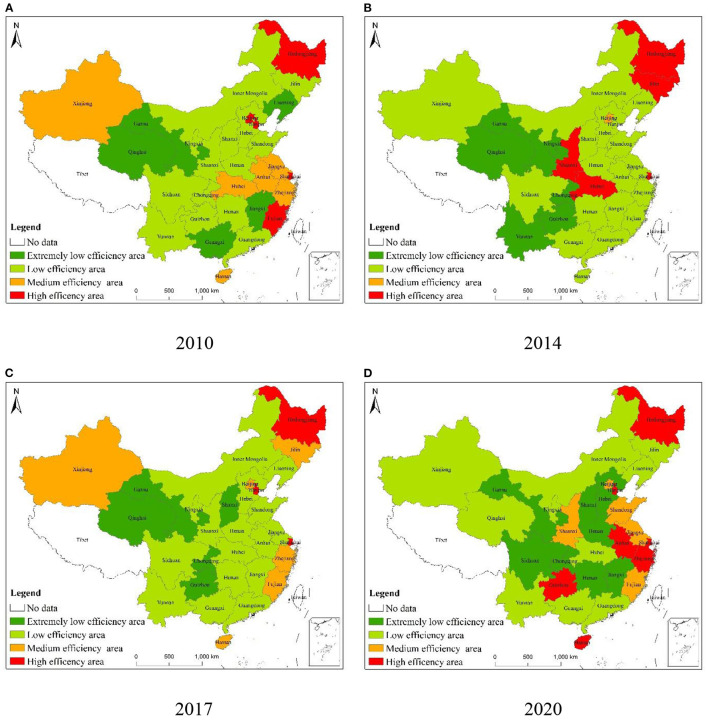
Spatial distribution of carbon emission efficiency of pig farming in China in some years **(A–D)**.

According to the carbon emission efficiency values of pig farming in China from 2010 to 2020, ArcGIS was used to draw the spatial distribution of carbon emission efficiency of pig farming in 30 provinces (autonomous regions and municipalities) nationwide, as shown in Figures 3A–D.

In 2010, Shanghai and Beijing in the eastern region were in the high and medium-efficiency zones respectively, while all other cities were in the low-efficiency zone. In the central region, Jilin, Heilongjiang, and Hubei were in the high-efficiency zone, while other cities were in the low-efficiency zone. In the western region, no city was in the medium-efficiency zone, with only Inner Mongolia, Xinjiang, Sichuan, and Shaanxi in the low-efficiency zone. All the other cities in the region were in a very low-efficiency zone. In 2014, the number of provinces in medium-efficiency zones increased from 1 to 5, and Xinjiang, Zhejiang, Fujian, and Hainan underwent a shift from low-efficiency zone to medium-efficiency zone. In the eastern region, the number of high-efficiency zones increased by one and the carbon emission efficiency of Tianjin went from a low-efficiency zone to a high-efficiency zone. The number of medium-efficiency zones also increased by four provinces, whereas the others were still located in the low-efficiency zones. In the central region, only Heilongjiang remained in the high-efficiency zone while other provinces were still in the low-efficiency zone. Jilin in the central region changed from the high-efficiency zone to the medium-efficiency zone and Hubei in the central region also changed from the high-efficiency zone to the low-efficiency zone.

From 2014 to 2017, the number of provinces in the medium efficiency zone increased from 5 to 6, while the number of provinces in the very low-efficiency zone increased from 6 to 9. Most of them were in the central and western regions, such as Sichuan, Hunan, Jiangxi, Henan, etc. In the eastern region, except for Hebei, Liaoning, and Guangdong which were in the very low-efficiency and low-efficiency zones, the remaining provinces were located in the medium and high-efficiency zones. It's also worth mentioning that the number of provinces in the high-efficiency zone reached five, so the efficiency of the eastern region has been improving faster. Sichuan, Hunan, Jiangxi, and Henan in the central region changed from low-efficiency areas to very low-efficiency areas in 2014, while Anhui province in the central region evolved from low-efficiency areas to high-efficiency areas. In 2020, the eastern region still has the highest number of provinces with high efficiency, while the central region has no province in high-efficiency areas. In the central region, there were two provinces, Anhui and Hubei in medium-efficiency areas and only Jiangxi with a very low-efficiency. Although the carbon emission efficiency of the western region was not as high as that of the eastern and central regions, the overall carbon emission efficiency has improved. For example, Xinjiang has changed from a low-efficiency area to a high-efficiency area, while Sichuan and Chongqing have changed from a very low-efficiency area to a low-efficiency area.

From 2010 to 2020, the carbon emission efficiency of pig breeding in China has improved to a certain extent and we have seen the trend of “East > Middle > West.” The number of areas with very low efficiency increased first followed by a decrease. From 2010 to 2017, the number of areas with very low efficiency increased from 6 to 9 and in 2020, the number dropped back to 6, which transferred to the Great Northwest and the Great Southwest comprehensive economic zones. From 2010 to 2020, the number of low-efficiency areas decreased from 16 to 13 and we have seen a shift from the eastern to the central and western regions, from the south to the north. During the same period, the number of areas with medium efficiency increased from 1 to 6 and we have seen a shift to Beijing, Tianjin, and South China water network areas. High-efficiency areas showed a trend of “decrease—increase—decrease” pattern and transferred to Beijing, Tianjin, Shanghai, and other areas with higher economic development levels.

### 3.2. Regression analysis of factors affecting carbon emission efficiency of pig farming

The mixed Tobit regression and random panel Tobit regression scores were applied to the model using STATA 14 software and the random panel Tobit model was selected by LR test to determine the direction of influence and the degree of influence of each influencing factor. The results are shown in Table 5.

**Table 5:** Tobit model estimation results.

**Variables**	**Hybrid Tobit regression**		**Stochastic panel Tobit regression**	

	**Estimated** **coefficient**	***p*** **-value**	**Estimated** **coefficient**	***p*** **-value**
*LS*	2.77000	0.18500	−0.73600^**^	0.67600
*PLI*	−0.00451	0.40200	0.00564	0.50700
*SG*	−7.40300^*^	0.09600	−5.04100^*^	0.05400
*BS*	0.15700	0.20500	0.35500^***^	0.00100
*JT*	0.23300^**^	0.03100	0.21100^**^	0.00200
*C*	0.41600^***^	0.00000	0.33100^***^	0.00000
*LR* 244.11 (*p* = 0.000)				

^*^, ^**^, ^***^indicate significance at the 10, 5, and 1% levels, respectively; *C* is the constant term, and *LR* is the likelihood ratio test value.

From the regression results, the regression coefficient value of food resources is −0.736 and it shows a significance at 0.05 level, implying that food resources have a significant negative correlation with carbon emission efficiency. For every one-unit increase in food resources, the carbon emission efficiency of pig farming will decrease by 0.736 units. The regression coefficient value of the production layout index is 0.00564 and the *p*-value is 0.507, implying that the production layout index has a non-significant positive influence on carbon emission efficiency. The carbon emission efficiency increases by 0.00113 units for each unit of increase in production layout index, the regression coefficient value of market size is −5.401 and it shows a significance of 0.1 level, implying that market size has a significant negative influence on carbon emission efficiency.

The regression coefficient value of the comparative advantage of the pig industry is 0.355 and it shows a significance at 0.01 level, which means that the comparative advantage of the pig industry has a significant positive influence on the carbon emission efficiency. With each unit increase in the comparative advantage of the pig industry, the carbon emission efficiency will increase by 0.355 units. The regression coefficient value of transportation accessibility is 0.211 and it shows a significance at 0.05 level, implying that transportation accessibility has a significant positive influence on carbon emission efficiency. With every one unit of increase in transportation accessibility, the carbon emission efficiency will increase by 0.211 units.

## 4. Discussion

### 4.1. Spatial and temporal characteristics of carbon emission efficiency of pig farming

#### 4.1.1. Time-series characteristics of carbon emission efficiency of pig farming in China

From 2010 to 2020, the carbon emission efficiency of pig farming in China roughly exhibited an “M” type growth trend with three different stages. From 2010 to 2015, the fluctuation of carbon emission efficiency of pig farming in China was in a stable and slow growth state, with little changes in the ratio of input to output. From 2015 to 2020, the carbon emission efficiency of China's pig farming showed an inverted M-shape trend. In 2015, the State promulgated the Regulations on Prevention and Control of Pollution from Livestock and Poultry Farming, which have put forward the requirement that the livestock and poultry farming industry should achieve sustainable development and minimize environmental pollution caused by livestock and poultry farming. As a result, various provinces (autonomous regions and municipalities) have actively responded to the national policies and they have taken measures to improve carbon emission efficiency and the ecological environment.

Although the carbon emission efficiency of China's pig farming decreased from 2015 to 2016, it rose back sharply from 2016 to 2018. In 2018, the outbreak of African swine fever (ASF) led to a significant decline in the number of pig farms. Hence the income of pig farmers and the number of people engaged in pig farming have also been severely impacted. The significant reduction in the supply of pigs led to an imbalance of demand and supply in the pork market, leading to a sharp rise in the price of pork. When the price of pork rose, consumers reduced their consumption of pork, resulting in a decline in output value and a significant decline in input and output. From 2018 to 2019, the carbon emission efficiency of pig farming in China decreased significantly from 0.601 to 0.406. After ASF was effectively controlled, the carbon emission efficiency of China's pig breeding in 2019–2020 improved somehow, but the improvement was not very significant due to the outbreak of COVID-19.

#### 4.1.2. Spatial characteristics of carbon emission efficiency of pig farming in China

Overall, the carbon emission efficiency of pig farming in all provinces (autonomous regions and municipalities) has improved to a certain extent from 2010 to 2019 and the local government of all provinces (autonomous regions and municipalities) have taken corresponding measures to improve carbon emission efficiency at source by implementing the concept of green development. From the regional perspective, the carbon emission efficiency value of China's pig farming showed a trend of “East > Middle > West” during 2010–2019. Moreover, a trend has been seen that the distribution of very low-efficiency areas and low-efficiency areas has transformed from eastern to central and western China. Also, the same trend has been seen from the southern part to the northern part of China with medium high-efficiency areas shifting to the eastern and southern parts of China.

The eastern region is mostly in the front of the country in terms of economic development state and potential, high degree of pig industry intensification, early development of pig scale, rich talent resources and mature production and breeding technology, high degree of effective transformation of animal husbandry resources, and large output to input ratio. At the same time, the development of pig farming in economically developed areas happened much earlier and the environmental regulation policies have been implemented earlier and more strictly. Hence, the pollutant emission management ability is stronger and the carbon emission efficiency is higher. With economic development, pork production is more influenced by the regional economic level, and economically developed areas gradually reduce pig farming. Traditional pig breeding areas, such as Zhejiang and Guangdong, have moved to Sichuan, Hubei, and Guizhou, which are the main producing areas of pig breeding currently.

In the central region, Henan is one of the ten main pig farming areas in China. Besides Henan, the traditional pig breeding areas are in the southern water network as well as Jilin and Heilongjiang in the northeast. These regions have a lower level of economic development with a more developed livestock industry, less intensive pig industry, lower level of pig breeding technology know-how, and less capability in pollution management. As a result, the carbon emission efficiency of pig breeding is lower than that of the eastern region. The central region is rich in grain resources and Henan, as the main producing area of corn and wheat, has abundant feed resources. That translates to low production cost, high scale and organization of pig farming, and a relatively developed pig processing industry, which has gathered many well-known domestic meat processing enterprises such as Shuanghui, Delis, and Jinluo. Jilin and Heilongjiang provinces are also China's main grain-producing areas, the agricultural labor force is rich in these two provinces and their breeding technology has certain capital and geographic advantages. These advantages will attract external investment in the pig industry from Beijing, Tianjin, and Shanghai, the Yangtze River Delta, and the Pearl River Delta.

However, the pig breeding technology in the central region is not mature and the pollution emissions in the breeding process have not been well-controlled, resulting in low carbon emission efficiency (31). The western region is greatly influenced by the level of economic development (32). Moreover, the development mode of animal husbandry is scattered and small with poor scale efficiency. As a result, the pollution surface is wide and scattered and governance is difficult (2, 33), leading to a low level of carbon emission efficiency.

### 4.2. Analysis of factors affecting carbon emission efficiency of pig farming

Since ancient times, there has been an old saying that “pigs and grains secure the world,” which shows that in the process of national economic development, pig farming and grain industry as two basic industries play an important role (34). As a grain-consuming industry, pig farming is based on grain production (especially corn industry and soybean industry), and from the perspective of the industrial chain, one of the most crucial constraints to the development of pig farming is the supply of upstream feeding materials (35). Therefore, pig farming and grain production are closely related. Pig farming is a grain-consuming livestock industry, which consumes a large amount of corn and other grain crops. The introduction of a market mechanism makes it possible to allocate resources to maximize production efficiency and concentrate on areas with rich grain resources. The more abundant the food resources are, the easier it is to become the main production area for pig farming. However, the economic development level of those areas is relatively low generally. The result is, for those more developed areas of the livestock industry, there're more carbon emissions from pig farming so the carbon emission efficiency is relatively low.

The pig production layout index is the performance of regional centralization and concentration of pig farming, which integrally reflects the regional distribution and the changes in the scale of pig farming. The production layout index of pigs is also subject to various factors, such as non-agricultural employment opportunities, science and technology, and economic factors. The higher the production layout index of pigs in a region, the more concentrated pig farming in the region is. Hence, it's much easier for pig farming to form an economy of scale. However, the results of the model regression show that the effect on carbon emission efficiency is not significant, indicating that the production layout index of pigs does not have a significant effect on the carbon emission efficiency of pig farming.

With the continuous promotion of market-oriented reform, the market gradually becomes the decisive force for resource allocation so the market demand is becoming more and more obvious for the development of pig farming. China is the world's number one pork-consuming country and pork is the most consumed meat product. With the improvement of income level, people will further increase the consumption of pork, thus stimulating farmers to increase the amount and scale of pig slaughter, which will constantly affect the layout and change of pig farming areas. Regions with large market scales will have more demand for the consumption of pork than those in other regions. As a result, it stimulates more farmers to increase the breeding of pigs. Through regression analysis, market scale and carbon emission efficiency show a negative correlation, and regions with large market scale will have higher breeding volume and lower carbon emission efficiency than those regions with small market scale.

If the benefits generated by pig farming in each region have obvious advantages over agricultural production, more farmers and enterprises will be attracted to increase their investment in scaling up pig farming as well as technological advancement. Through the regression results, the comparative advantage of the pig industry has a positive influence over carbon emission efficiency, which indicates that regions with obvious comparative advantage in the pig industry attract more capital and technology to that region. The introduction of technology can improve pig breeding, pig rearing environment, feed, and grain types, hence improving pig rearing efficiency and reducing carbon emissions generated in the pig breeding process. At the same time, the introduction of capital can make farmers increase their investment in the intestinal fermentation and manure excretion aspects of the pig breeding process, thus reducing carbon emissions and improving carbon emission efficiency.

The separation of pig farmers and consumers and the differentiation of production and consumption areas require convenient transportation facilities to ease the contradiction between supply and demand and to reduce transportation costs. Through transportation facilities, modern production factors such as capital, technology, and information can also be quickly transferred to producers, thus improving production efficiency. Convenient transportation conditions have a great positive impact on the distribution of regional feeds, transportation, and sales of pigs. Developed transportation systems not only facilitate sales, but also transfers information and technology to pig farmers timely, which facilitates farmers to understand relevant technical know-how to improve pig farming technology. Hence the carbon emission can be better controlled in the pig farming process, thus improving carbon emission efficiency (31).

## 5. Conclusions

By comparing the spatial and temporal characteristics of the carbon emission efficiency of pig farming in 30 provinces (autonomous regions and municipalities) in China during the study period, it was found that the carbon emission efficiency of pig farming in China showed an M-shape growth trend. Moreover, the trends of the three regions identified were the same as the changing trend of carbon emission efficiency of pig farming in China. Through horizontal comparison, we found that the carbon emission efficiency of the three regions of the eastern, middle, and western parts of China showed the trend of “East > Middle > West” and the very low-efficiency areas and low-efficiency areas shifted from eastern to middle and western part of China. At the same time, areas with middle and high efficiency shifted to the east.

For pig farming, changes in various influencing factors will contribute to changes in the carbon emission efficiency of pig farming. The regression analysis found that the comparative advantage of the pig industry and transportation accessibility had a significant positive influence on the carbon emission efficiency of pig farming. The proportion of food resources and market size had a significant negative correlation on carbon emission efficiency. The production layout index had no significant influence on the carbon emission efficiency generated by pig farming.

## Data availability statement

The original contributions presented in the study are included in the article/supplementary material, further inquiries can be directed to the corresponding author.

## Author contributions

Conceptualization, resources, writing—original draft, project administration, and funding acquisition: HG. Methodology, software, validation, formal analysis, and investigation: HG and SL. Data curation: SL. Writing—review and editing: SL, HG, and SX. Visualization: CP. Supervision: QL. All authors have read and agreed to the published version of the manuscript.

## Footnotes

^1^“China Statistical Yearbook” (https://data.cnki.net/yearbook/Single/N2022110021).

^2^“China Rural Statistical Yearbook” (https://data.cnki.net/yearbook/Single/N2021120010).

^3^“China Rural Statistical Yearbook” (https://data.cnki.net/yearbook/Single/N2021120010).
